# Supplementary material for: FoxO3 an important player in fibrogenesis and therapeutic target for idiopathic pulmonary fibrosis
Source: EMBO Mol Med. 2017 Dec 7;10(2):276–93. doi: 10.15252/emmm.201606261 (PMC5801513; doi:10.15252/emmm.201606261)
Supplement: Supplementary file 1 — Appendix [file EMMM-10-276-s001.docx]

**Appendix**

**Table of Content**

**Appendix Fig S1**

**Appendix Fig S2**

**Appendix Fig S3**

**Appendix Fig S4**

**Appendix Fig S5**

**Appendix Fig S6**

**Appendix Fig S7**

**Appendix Fig S8**

**Appendix Fig S9**

**Appendix Fig S10**

**Appendix Fig S11**

**Appendix Fig S12**

**Appendix Fig S13**

**Appendix Fig S14**

**Appendix Fig S15**

**Appendix Table S1**

**Appendix Table S2**

**Appendix Table S3**


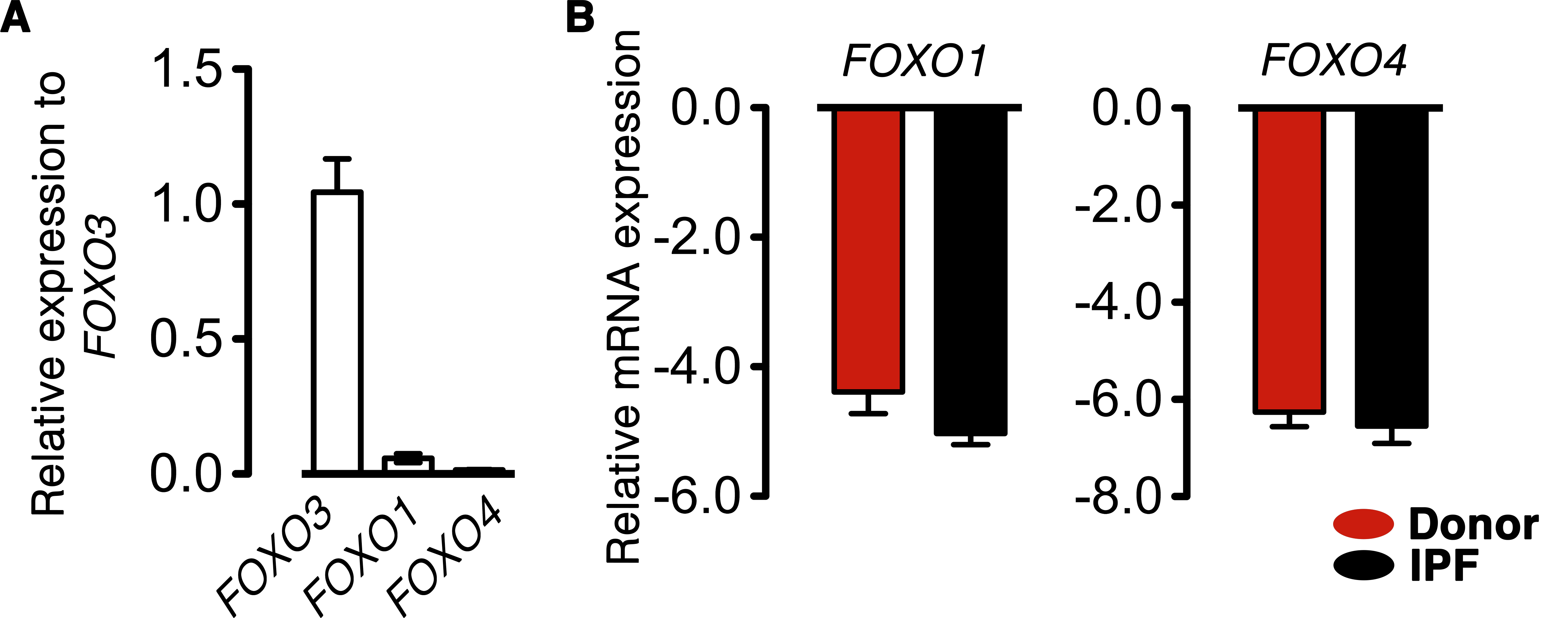


**Fig S1. FOXO1 and FOXO4 expression in N-HLF and IPF-HLF**

A mRNA expression analysis of *FOXO1*, *3* and *4* genes by qPCR in N-HLF (isolated from healthy donor). Expression levels are relative to *FOXO3*. *FOXO3* expression has been considered as 1. Bar indicates mean ± S.E.M (n=7).

B mRNA expression analysis of *FOXO1* and *FOXO4* in N-HLF and IPF-HLF by qPCR (n=7/group).

**
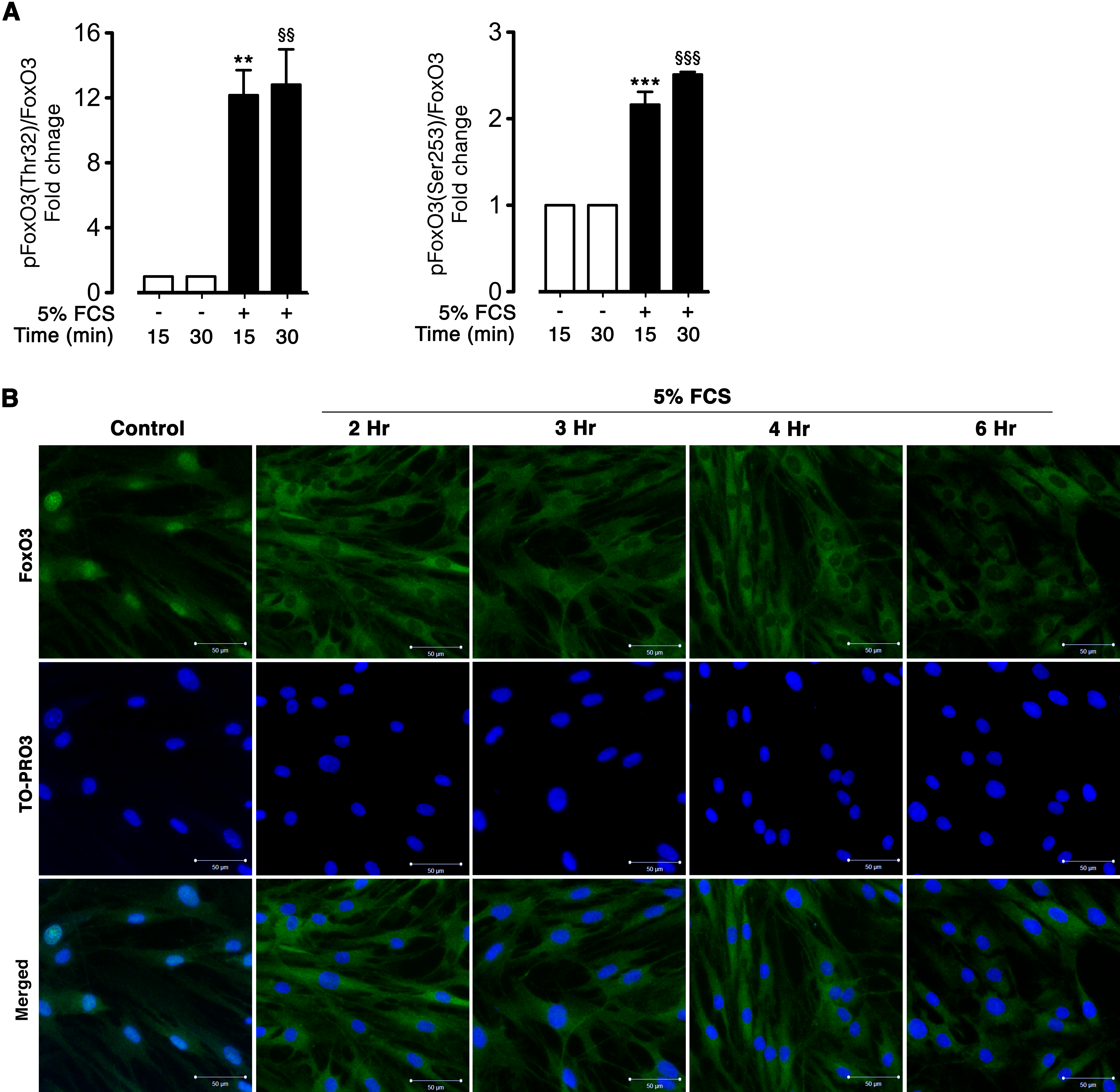
**

**Fig S2. FCS induces FoxO3 phosphorylation and nuclear exclusion of FoxO3.**

A Densitometry quantitation ratio of p-FoxO3 (Thr32) (left panel) and p-FoxO3 (Ser253) (right panel) in serum starved (48 hours) N-HLF (n=3) that were stimulated without/with 5% FCS as indicated. Quantification is represented as a fold change to control (time corresponded non-stimulated cells). Data were using repeated measures ANOVA, **p <0.01, ***p <0.001 versus control 15 min, ^§§^p <0.01, ^§§§^p <0.001 versus control 30 min.

B ICC of FoxO3 in N-HLF that were serum starved for 48 hours, and stimulated with 5% FCS as indicated. Control image panel represents cells that left non-stimulated for 6 hours. TO-PRO3 (blue) was used to label nuclei. FoxO3 and TO-PRO3 images were overlaid to visualize nuclear and cytoplasmic localization of FoxO3. Images are representative of n=3. Scale bar=50μm.

**
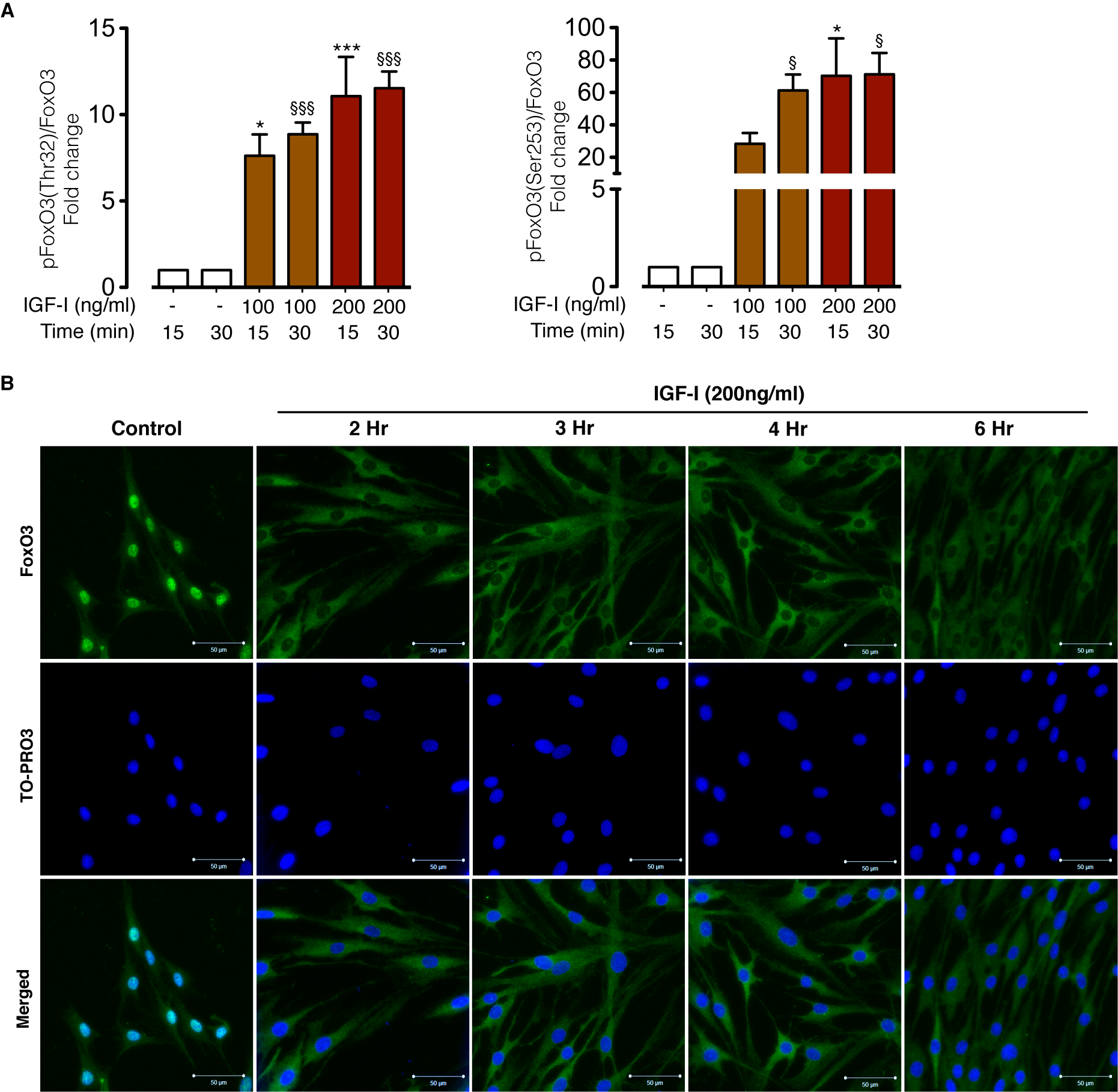
**

**Fig S3. IGF-I induces FoxO3 phosphorylation and nuclear exclusion of FoxO3.**

A Densitometry quantitation ratio of p-FoxO3 (Thr32) (left panel) and p-FoxO3 (Ser253) (right panel) in serum starved (48 hours) N-HLF (n=3) that were stimulated without/ with IGF-I as indicated. Quantification is represented as a fold change to control (time corresponded non-stimulated cells). Data were using repeated measures ANOVA, *p <0.05, **p <0.01, ***p <0.001 versus control 15 min, ^§§^p <0.01, ^§§§^p <0.001 versus control 30 min.

B ICC of FoxO3 in N-HLF that were serum starved for 48 hours, and stimulated with IGF-I (200ng/ml) as indicated. Control image panel represents cells that left non-stimulated for 6 hours. TO-PRO3 (blue) was used to label nuclei. FoxO3 and TO-PRO3 images were overlaid to visualize nuclear and cytoplasmic localization of FoxO3. Images are representative of n=3. Scale bar=50μm.

**Fig S4. Various growth factors induce FoxO3 phosphorylation to similar extent in IPF-HLFs**

(A) Representative western blots of p-FoxO3 (Thr32), FoxO3 and GAPDH in serum starved (48hrs) IPF-HLF (n=3) that were stimulated with 5%FCS or PDGF-BB or IGF-1 as indicated for 30min. Densitometry quantified data of p-FoxO3 (Thr32) to FoxO3 expression ratios, represented as a fold change to non-stimulated cells. Data were analyzed using repeated-measures one-way ANOVA *p<0.05, **p<0.01, ***p<0.001 versus vehicle treated cells.

**Fig S5. Various growth factors induce FoxO3 phosphorylation to similar extent in N-HLFs**

Representative western blots of p-FoxO3 (Thr32), FoxO3 and GAPDH in serum starved (48hrs) N-HLF (n=3) that were stimulated with 5%FCS or PDGF-BB or IGF-1 as indicated for 30min. Densitometry quantified data of p-FoxO3 (Thr32) to FoxO3 expression ratios, represented as a fold change to non-stimulated cells. Data were analyzed using repeated-measures one-way ANOVA *p<0.05, **p<0.01, ***p<0.001 versus vehicle treated cells.

**Fig S6. Passaging of HLFs do not effect FoxO phophorylation by growth factors**

Western blots of p-FoxO3 (Thr32), FoxO3 and GAPDH in serum starved (48hrs) (A) N-HLF or (B) IPF-HLF that were stimulated with 5%FCS or PDGF-BB or IGF-1 over different passages (P3 to P5) as indicated.


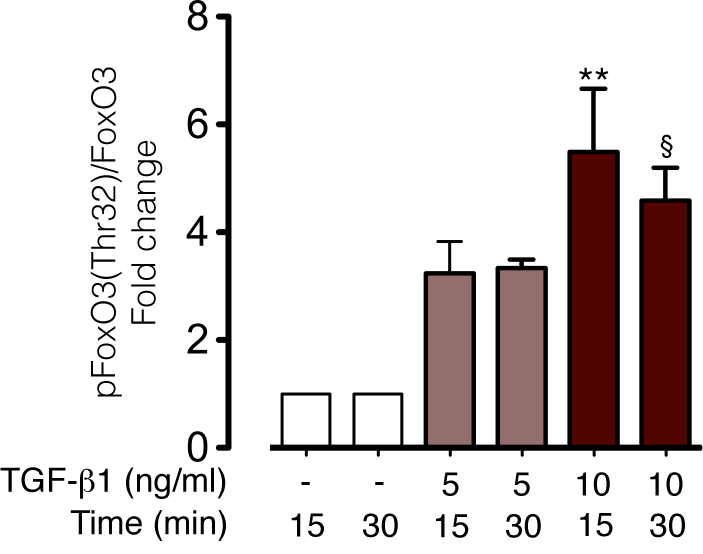


**Fig S7. TGF-β1 induces FoxO3 phosphorylation.**

Densitometry quantitation ratio of p-FoxO3 (Thr32) in serum starved (48 hours) N-HLF (n=3) that were stimulated without/ with TGF-β1 as indicated. Quantification is represented as a fold change to control (time corresponded non-stimulated cells). Data were using repeated measures ANOVA, *p <0.05, **p <0.01 versus control 15 min, ^§^p <0.05, ^§§^p <0.01 versus control 30 min.


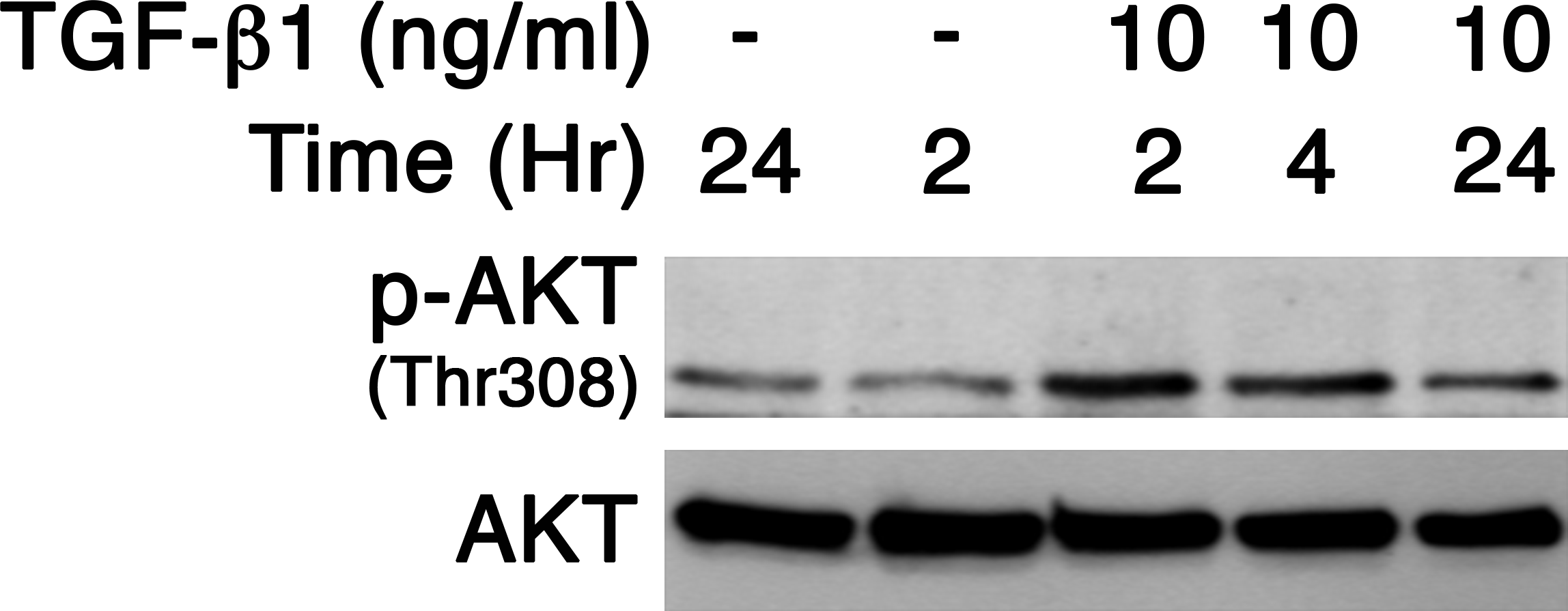


**Fig S8. TGF-β1 induces AKT phosphorylation.**

Western blots of p-AKT (Thr308) in serum starved (48 hours) N-HLF (n=3) that were stimulated with/without TGF-β1as indicated.


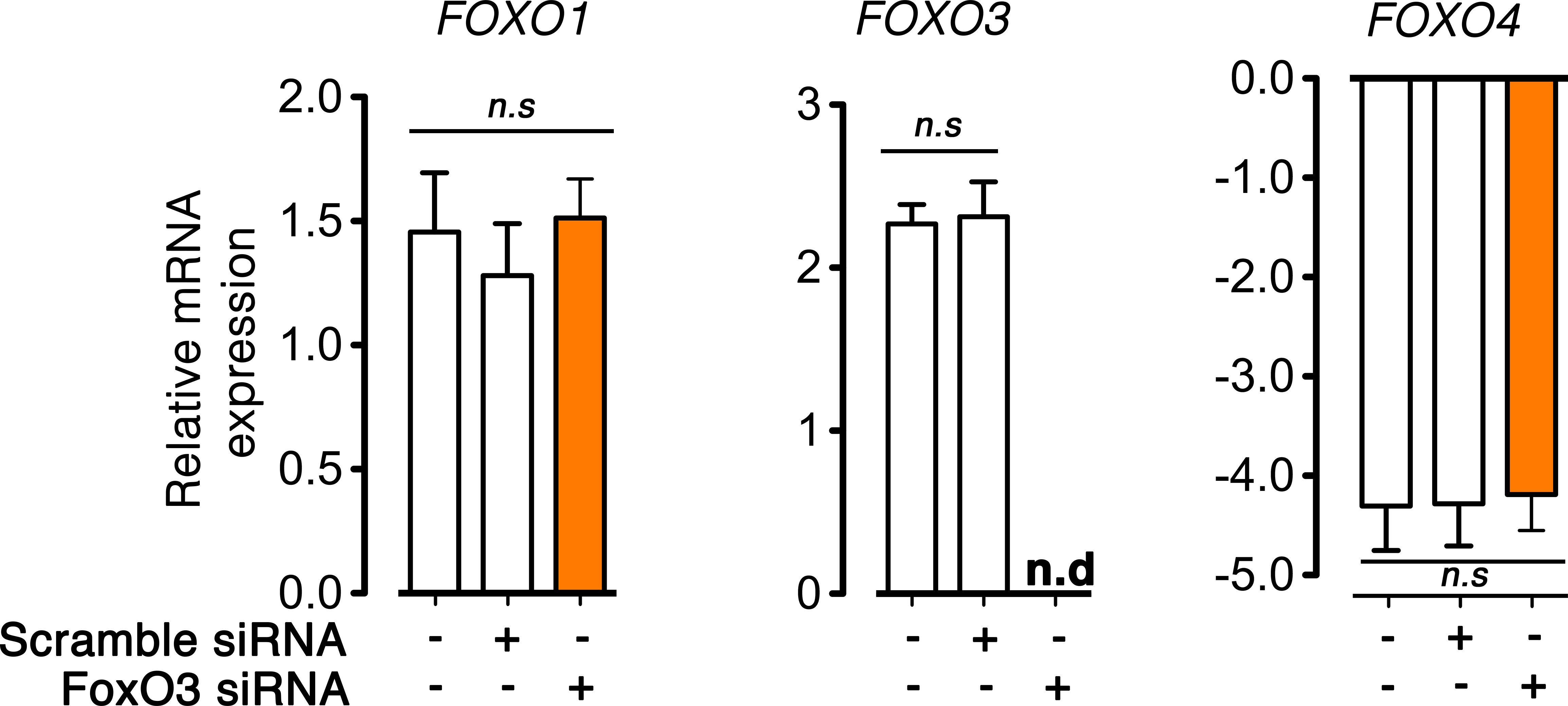


**Fig S9. Specific knockdown of *FOXO3* in primary human lung fibroblasts.**

mRNA expression analysis of *FOXO1*, *FOXO3* and *FOXO4* by qPCR. n=2 and duplicate were performed for each condition. N-HLF were transfected with scramble siRNA or FoxO3 siRNA. An additional control was added in which the cells were untransfected. 6 hours after transfection, cells were serum starved for 60 hours followed by RNA isolation. n.d= not detected. Data were analyzed using one-way ANOVA, n.s= not significant.

**Fig S10. FOXO3 regulates *MYOCARDIN* expression**

N-HLFs (n=3) were transfected with scramble siRNA or FoxO3 siRNA. mRNA expression of MYOCD was analyzed by qPCR. Data were analyzed using Student’s t test, *p<0.05, **p<0.01, ***p<0.001 versus scramble siRNA group.

**Fig S11. Fibroblast specificity of *Foxo3_f.b_^-/-^* mice**

Fibroblasts and alveolar type II epithelial cells were treated were isolated from *Foxo3****_f.b_****^-/-^* and WT littermates. mRNA expression analysis of Foxo3 by qPCR (n=3-4/5 per group). Data were analyzed using Student’s t test, *p<0.05, **p<0.01, ***p<0.001 versus WT group. Immunofluorescence staining was performed on WT and *Foxo3_f.b_^-/-^* mice lung sections (saline and bleomycin instilled) using FoxO3 and α-SMA antibodies. (C) Representative pictographs showing FoxO3 staining in green, with α-SMA stained in red. DAPI was used as nuclear stain. Scale = 50μm.


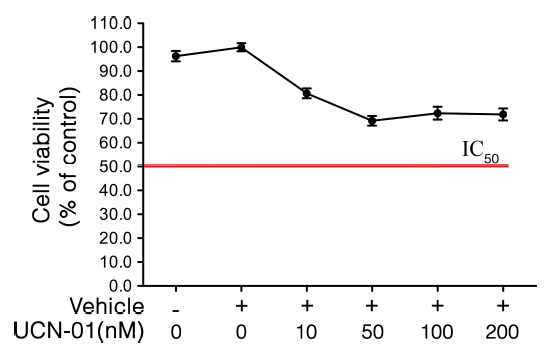


**Fig S12.** **MTT assay of UCN-01**

Human lung fibroblasts from controls (N-HLF) were serum starved for 48 hrs. Then, cells were treated with UCN-01 (10, 50, 100 and 200nM) or vehicle (DMSO) or left untreated and cell viability was assessed by MTT assay after 24 hrs. Data are represented as percentage of control; vehicle treated cells. Bars indicate means± S.E.M (n=3). Red line indicates IC_50_.

**Fig S13. UCN-01 inhibits FoxO3 nuclear exclusion in FCS, stimulated N-HLF.**

N-HLFs (n=3) were serum starved for 48hrs followed by stimulation with 5% FCS for 6hrs in medium containing UCN-01 (50nM) as indicated. Representative western blots of p-FoxO3 (Thr32), FoxO3, LAMIN B1 and TUBA1B from the cytoplasmic (C) and nuclear fractions (N). Densitometry quantified data of FoxO3 to p-FoxO3 (Thr32) and the ratio was normalized to TUBA1B for cytoplasmic fraction and LAMIN B1 for nuclear fraction. Expression ratios are represented as a fold change to cytosolic fraction of 5% FCS treated cells. Data were analyzed using Student’s t test, *p<0.05, **p<0.01, ***p<0.001.

**Fig S14. Wortmanin inhibits AKT and FoxO3 phosphorylation induced by TGFβ**

Representative western blots of p-FoxO3 (Thr32), FoxO3, p-AKT (Thr308), AKT and GAPDH in serum starved (48hrs) IPF-HLF (n=3) that were stimulated with TGF-β1 for 4hrs in medium containing Wortmanin (Wort.) (250nM or 500nM) or UCN-01 (50nM) as indicated. Densitometry quantified data of p-FoxO3 (Thr32) to FoxO3 and p-AKT (Thr308) to AKT expression ratios, represented as a fold change to non-stimulated cells. Data were analyzed using repeated-measures one-way ANOVA *p<0.05, **p<0.01, ***p<0.001 versus TGFβ treated cells.


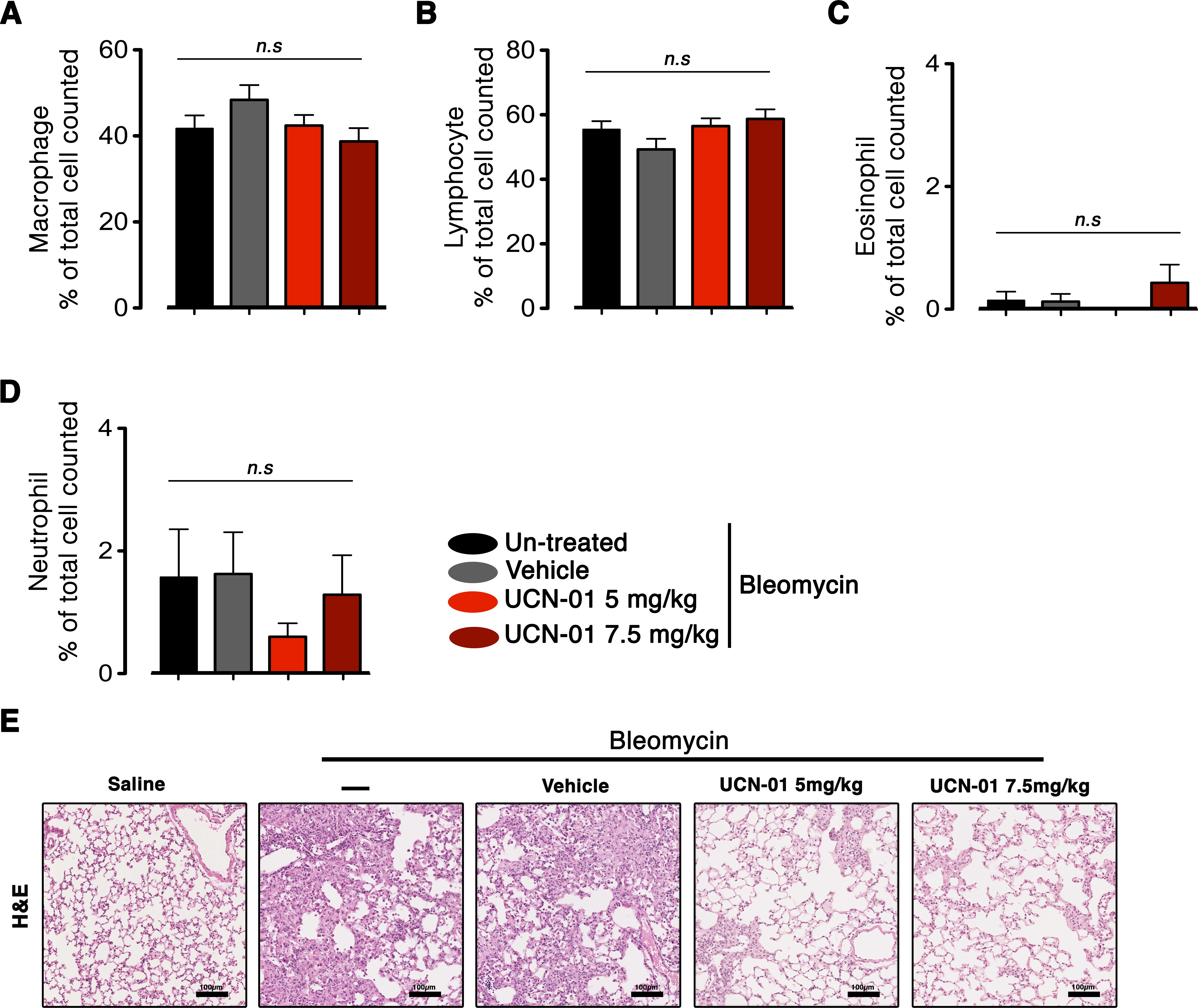


**Fig S15. UCN-01 treatment does not mediate its effect via changing the inflammatory responses.**

A-E Number of inflammatory cells in BALF (in percentage). (A) Macrophages (B) Lymphocyte (C) Eosinophil and (D) Neutrophil Bleomycin non- (n=7 mice), Bleomycin vehicle-treated (n=8 mice), UCN-01 5mg/kg treated (n=10 mice) and UCN-01 7.5mg/kg.bw treated mice (n=7 mice). Data were analyzed using one-way ANOVA n.s=not significant. (E) Representative H&E staining of mice lungs at higher magnification. Scale bar=100µm.

**Table S1**. **List of primers.**

| **Gene** | **Species** | **Primers 5’- 3’** |
| --- | --- | --- |
| *ACTA2* | Human | F: AGA TCA AGA TCA TTG CCC C  R: TTC ATC GTA TTC CTG TTT CG |
| *COL1A1* | Human | F: AAG CGA GGA GCT CGA GGT GAA C  R: TTG GCA CCA GGC AGA CCA GCT T |
| *COL3A1* | Human | F: TGG GAG AAA TGG TGA CCC TGG  R: CCA GGA TAG CCT GCG AGT CCT |
| *FOXO1* | Human | F: CAA TGG AAC ATC CCA AGA AG  R: CAA GGA GAG GCC AAC TGT AA |
| *FOXO3* | Human | F: GCT GAA GGA TCA CTG AGG AA  R: CAG TCT CTG CTG GGT TAG GA |
| *FOXO4* | Human | F: GTC CCC TCA TTC CTT CAA CT  R: GCC ACA CAC ACT TCC ACA TA |
| *HPRT* | Human | F: TGA CAC TGG CAA AAC AAT GCA  R: GGT CCT TTT CAC CAG CAA GCT |
| *Foxo1* | Mouse | F: GAT AAG GGC GAC AGC AAC AG  R: TTG AGC ATC CAC CAA GAA CTC |
| *Foxo3* | Mouse | F: CGC TGT GTG CCC TAC TTC  R: CCC GTG CCT TCA TTC TGA |
| *Foxo4* | Mouse | F: GGA TGG TGA GGG ACT GGA CT  R: GAG GAA AAG TGG ATA AGG AC AGG |
| *Hprt* | Mouse | F: GCT GAC CTG CTG GAT TAC AT  R: TTG GGG CTG TAC TGC TTA AC |

**Table S2. List of antibodies.**

| **Antibody** | **Reactivity** | **Source** | **Dilution** | **Company** | **Catalog No.** |
| --- | --- | --- | --- | --- | --- |
| p-AKT | Human | Rabbit | 1:1000 | Cell Signaling | 4056 |
| Col1a | Human | Rabbit | 1:1000 | Meridian Life Science | T40777R |
| α-SMA | Human | Mouse | 1:500 | Sigma Aldrich | WH0000059M2 |
| FoxO1 | Human | Rabbit | 1:1000 | Novus | NB100-2312 |
| FoxO3 | Human | Rabbit | 1:500 | Epitomics | 2071-1 |
| FoxO3 | Mouse | Rabbit | 1:500 | Thermo Scientific | MA5-14932 |
| FoxO4 | Human/Mouse | Rabbit | 1:500 | Cell Signaling | 9472 |
| p-FoxO3-Ser253 | Human | Rabbit | 1:300 | Cell Signaling | 9466 |
| p-FoxO1 Thr24/p-FoxO3 Thr32 | Human/Mouse | Rabbit | 1:500 | Cell Signaling | 9564 |
| β-actin | Mouse | Mouse | 1:4000 | Sigma Aldrich | A5316 |
| GAPDH | Human/Mouse | Mouse | 1:4000 | Novus | NB300-221 |

**Table S3. Statistical analysis.**

**Fig. 1**

| **Groups compared** | **Summary** | **p value** | **Statistical test** |
| --- | --- | --- | --- |
| Fig.1a Donor Vs IPF No FCS | ***** | 0.0237 | Student’s t test |
| Fig.1a Donor Vs IPF 5%FCS | ******* | <0.0001 | Student’s t test |
| Fig. 1B Donor Vs IPF COL1A1 | ****** | 0.0078 | Student’s t test |
| Fig. 1B Donor Vs IPF COL3A1 | ***** | 0.0170 | Student’s t test |
| Fig. 1C Donor Vs IPF FOXO3 | ****** | 0.0095 | Student’s t test |
| Fig. 1D Donor Vs IPF FOXO3 |  | 0.08 | Student’s t test |
| Fig. 1F Donor Vs IPF pFOXO3Thr32/GAPDH | ***** | 0.0229 | Student’s t test |
| Fig. 1F Donor Vs IPF pFOXO3Ser 253/GAPDH | ***** | 0.0318 | Student’s t test |
| Fig. 1L control vs TGFβ1  Relative mRNA expression | ******* | 0.0005 | Unpaired t test |
| Fig. 1L control vs TGFβ1  FoxO3/GAPDH | ***** | 0.0330 | Unpaired t test |

**Fig. 2**

| **Groups compared** | **Summary** | **p value** | **Statistical test** |
| --- | --- | --- | --- |
| Fig.2A Scramble Vs FoxO3 siRNA | ***** | 0.0236 | One way ANOVA followed by Tukey’s test |
| Fig.2B Scramble Vs FoxO3 siRNA No FCS | ***** | 0.0193 | One way ANOVA followed by Tukey’s test |
| Fig.2B Scramble No FCS Vs Scramble siRNA FCS | ******* | <0.0001 | One way ANOVA followed by Tukey’s test |
| Fig.2B Scramble No FCS Vs FoxO3 siRNA FCS | ******* | <0.0001 | One way ANOVA followed by Tukey’s test |
| Fig.2B Scramble FCS Vs FoxO3 siRNA FCS | **§§§** | <0.0001 | One way ANOVA followed by Tukey’s test |
| Fig.2B Scramble siRNA (No IGF-1) Vs Scramble siRNA (IGF-1) | ******* | 0.0009 | One way ANOVA followed by Tukey’s test |
| Fig.2B Scramble siRNA (No IGF-1) Vs FoxO3 siRNA (IGF-1) | ******* | <0.0001 | One way ANOVA followed by Tukey’s test |
| Fig.2B FoxO3 siRNA (No IGF-1) Vs FoxO3 siRNA (IGF-1) | **n.s.** |  | One way ANOVA followed by Tukey’s test |
| Fig.2B Scramble siRNA (No PDGF-BB) Vs Scramble siRNA (PDGF-BB) | ******* | <0.0001 | One way ANOVA followed by Tukey’s test |
| Fig.2B Scramble siRNA (No PDGF-BB) Vs FoxO3 siRNA (PDGF-BB) | ******* | <0.0001 | One way ANOVA followed by Tukey’s test |
| Fig.2B FoxO3 siRNA (No PDGF-BB) Vs FoxO3 siRNA (PDGF-BB) | **§§§** | <0.0001 | One way ANOVA followed by Tukey’s test |
| Fig.2C Scramble siRNA Vs FoxO3 siRNA COL1A1 | ******* | <0.0001 | Student’s t test |
| Fig.2C Scramble siRNA Vs FoxO3 siRNA COL3A1 | ******* | <0.0001 | Student’s t test |
| Fig.2C Scramble siRNA Vs FoxO3 siRNA ACTA2 | ******* | <0.0001 | Student’s t test |

**Fig. 3**

| **Groups compared** | **Summary** | **p value** | **Statistical test** |
| --- | --- | --- | --- |
| Fig.3A Saline Vs Day14 | ***** | 0.0440 | Student’s t test |
| Fig.3A Saline Vs Day21 | ***** | 0.0335 | Student’s t test |
| Fig.3B Saline Vs Day14 FoxO3/Actb | ****** | 0.0019 | Student’s t test |
| Fig.3B Saline Vs Day21 FoxO3/Actb | ***** | 0.0327 | Student’s t test |
| Fig.3B Saline Vs Day21 pFoxO3/(Thr32)/FoxO3 | ***** | 0.0115 | Student’s t test |
| Fig.3B Saline Vs Day14 α-SMA/Actb | ***** | 0.0419 | Student’s t test |
| Fig.3B Saline Vs Day21 α-SMA/Actb | ***** | 0.0176 | Student’s t test |
| Fig.3D WT saline Vs WT Bleo | ****** | 0.0035 | One way ANOVA followed by Tukey’s test |
| Fig.3D WT saline Vs Foxo3^-/-^ Bleo | ******* | <0.0001 | One way ANOVA followed by Tukey’s test |
| Fig.3D WT saline Vs Foxo3_f.b_^-/-^ Bleo | ******* | <0.0001 | One way ANOVA followed by Tukey’s test |
| Fig.3D WT Bleo Vs Foxo3_f.b_^-/-^ Bleo | **§§§** | 0.0009 | One way ANOVA followed by Tukey’s test |
| Fig.3E WT saline Vs WT Bleo | ******* | <0.0001 | One way ANOVA followed by Tukey’s test |
| Fig.3E WT saline Vs Foxo3^-/-^ Bleo | ******* | <0.0001 | One way ANOVA followed by Tukey’s test |
| Fig.3E WT saline Vs Foxo3_f.b_^-/-^ Bleo | ******* | <0.0001 | One way ANOVA followed by Tukey’s test |
| Fig.3E WT Bleo Vs Foxo3^-/-^ Bleo | **§** | 0.0122 | One way ANOVA followed by Tukey’s test |
| Fig.3E WT Bleo Vs Foxo3_f.b_^-/-^ Bleo | **§§§** | 0.0002 | One way ANOVA followed by Tukey’s test |
| Fig.3F WT saline Vs WT Bleo | ***** | 0.0203 | One way ANOVA followed by Tukey’s test |
| Fig.3F WT saline Vs Foxo3^-/-^ Bleo | ******* | <0.0001 | One way ANOVA followed by Tukey’s test |
| Fig.3F WT saline Vs Foxo3_f.b_^-/-^ Bleo | ******* | <0.0001 | One way ANOVA followed by Tukey’s test |
| Fig.3F WT Bleo Vs Foxo3^-/-^ Bleo | **§** | 0.0173 | One way ANOVA followed by Tukey’s test |
| Fig.3F WT Bleo Vs Foxo3_f.b_^-/-^ Bleo | **§§§** | 0.0004 | One way ANOVA followed by Tukey’s test |
| Fig.3G WT saline Vs WT Bleo | ******* | <0.0001 | One way ANOVA followed by Tukey’s test |
| Fig.3G WT saline Vs Foxo3^-/-^ Bleo | ******* | <0.0001 | One way ANOVA followed by Tukey’s test |
| Fig.3G WT saline Vs Foxo3_f.b_^-/-^ Bleo | ******* | <0.0001 | One way ANOVA followed by Tukey’s test |
| Fig.3G WT Bleo Vs Foxo3^-/-^ Bleo | **n.s.** | 0.0583 | One way ANOVA followed by Tukey’s test |
| Fig.3G WT Bleo Vs Foxo3_f.b_^-/-^ Bleo | **§§§** | 0.0099 | One way ANOVA followed by Tukey’s test |
| Fig.3I WT saline Vs WT Bleo | ******* | <0.0001 | One way ANOVA followed by Tukey’s test |
| Fig.3I WT saline Vs Foxo3^-/-^ Bleo | ******* | <0.0001 | One way ANOVA followed by Tukey’s test |
| Fig.3I WT saline Vs Foxo3_f.b_^-/-^ Bleo | ******* | <0.0001 | One way ANOVA followed by Tukey’s test |
| Fig.3I WT Bleo Vs Foxo3_f.b_^-/-^ Bleo | **§** | 0.0131 | One way ANOVA followed by Tukey’s test |

**Fig. 4**

| **Groups compared** | **Summary** | **p value** | **Statistical test** |
| --- | --- | --- | --- |
| Fig.4A Vehicle Vs UCN-01 (50nM) | ******* | <0.0001 | One way ANOVA followed by Tukey’s test |
| Fig.4A Vehicle Vs UCN-01 (100nM) | ******* | <0.0001 | One way ANOVA followed by Tukey’s test |
| Fig.4A Vehicle Vs UCN-01 (200nM) | ******* | <0.0001 | One way ANOVA followed by Tukey’s test |
| Fig.4B Vehicle Vs UCN-01 | ******* | <0.0001 | One way ANOVA followed by Tukey’s test |
| Fig.4C Vehicle Vs UCN-01 | ******* | <0.0001 | One way ANOVA followed by Tukey’s test |
| Fig.4D Vehicle (No TGFβ1) Vs Vehicle (TGFβ1) COL1A1 | ******* | 0.0004 | One way ANOVA followed by Tukey’s test |
| Fig.4D Vehicle (TGFβ1) Vs UCN-01 (TGFβ1) COL1A1 | ***** | 0.0268 | One way ANOVA followed by Tukey’s test |
| Fig.4D Vehicle (No TGFβ1) Vs Vehicle (TGFβ1) COL3A1 | ***** | 0.0362 | One way ANOVA followed by Tukey’s test |
| Fig.4D Vehicle (TGFβ1) Vs UCN-01 (TGFβ1) COL3A1 | ****** | 0.0018 | One way ANOVA followed by Tukey’s test |
| Fig.4D Vehicle (No TGFβ1) Vs Vehicle (TGFβ1) ACTA2 | ******* | <0.0001 | One way ANOVA followed by Tukey’s test |
| Fig.4D Vehicle (TGFβ1) Vs UCN-01 (TGFβ1) ACTA2 | ******* | <0.0001 | One way ANOVA followed by Tukey’s test |
| Fig.4E Vehicle (No TGFβ1) Vs Vehicle (TGFβ1) Col1/GAPDH | ***** | 0.0270 | One way ANOVA followed by Tukey’s test |
| Fig.4E Vehicle (TGFβ1) Vs UCN-01 (TGFβ1) Col1/GAPDH | ****** | 0.0019 | One way ANOVA followed by Tukey’s test |
| Fig.4E Vehicle (No TGFβ1) Vs Vehicle (TGFβ1) α-SMA /GAPDH | ***** | 0.0284 | One way ANOVA followed by Tukey’s test |
| Fig.4E Vehicle (TGFβ1) Vs UCN-01 (TGFβ1) α-SMA /GAPDH | ***** | 0.0254 | One way ANOVA followed by Tukey’s test |

**Fig. 5**

| **Groups compared** | **Summary** | **p value** | **Statistical test** |
| --- | --- | --- | --- |
| Fig.5A Vehicle (No FCS) Vs Vehicle (5%FCS) pFoxO3(Ser253)/FoxO3 | ***** | 0.0190 | One way ANOVA followed by Tukey’s test |
| Fig.5A Vehicle (5%FCS) Vs UCN-01 (5% FCS) pFoxO3(Ser253)/ FoxO3 | ***** | 0.0433 | One way ANOVA followed by Tukey’s test |
| Fig.5A Vehicle (5%FCS) Vs Wort. (5% FCS) pFoxO3(Ser253)/ FoxO3 | ***** | 0.0462 | One way ANOVA followed by Tukey’s test |
| Fig.5A Vehicle (No FCS) Vs Vehicle (5%FCS) pFoxO3(Thr32)/ FoxO3 | ***** | 0.0328 | One way ANOVA followed by Tukey’s test |
| Fig.5A Vehicle (5%FCS) Vs UCN-01 (5% FCS) pFoxO3(Thr32)/ FoxO3 | ***** | 0.0416 | One way ANOVA followed by Tukey’s test |
| Fig.5A Vehicle (5%FCS) Vs Wort. (5% FCS) pFoxO3(Thr32)/ FoxO3 | ***** | 0.0475 | One way ANOVA followed by Tukey’s test |
| Fig.5C Vehicle (No FCS) Vs Vehicle (5%FCS) pAKT (Thr308)/AKT | ***** | 0.0187 | One way ANOVA followed by Tukey’s test |
| Fig.5C Vehicle (5%FCS) Vs UCN-01 (5% FCS) pAKT (Thr308)/AKT | ***** | 0.0245 | One way ANOVA followed by Tukey’s test |
| Fig.5C Vehicle (5%FCS) Vs Wort. (5% FCS) pAKT (Thr308)/AKT | ***** | 0.0128 | One way ANOVA followed by Tukey’s test |
| Fig.5E Vehicle (No IGF-1) Vs Vehicle (IGF-1) pFoxO3(Thr32)/ FoxO3 | ******* | <0.0001 | One way ANOVA followed by Tukey’s test |
| Fig.5E Vehicle (IGF-1) Vs UCN-01 (IGF-1) pFoxO3(Thr32)/ FoxO3 | ***** | 0.0284 | One way ANOVA followed by Tukey’s test |
| Fig.5E Vehicle (IGF-1) Vs Wort. (IGF-1) pFoxO3(Thr32)/ FoxO3 | ******* | <0.0001 | One way ANOVA followed by Tukey’s test |
| Fig.5F Vehicle (No IGF-1) Vs Vehicle (IGF-1) pAKT (Thr308)/AKT | ****** | 0.0070 | One way ANOVA followed by Tukey’s test |
| Fig.5F Vehicle (IGF-1) Vs UCN-01 (IGF-1) pAKT (Thr308)/AKT | ***** | 0.0202 | One way ANOVA followed by Tukey’s test |
| Fig.5F Vehicle (IGF-1) Vs Wort. (IGF-1) pAKT (Thr308)/AKT | ****** | 0.0084 | One way ANOVA followed by Tukey’s test |
| Fig.5H Vehicle (No PDGF-BB) Vs Vehicle (PDGF-BB) pFoxO3(Ser253)/FoxO3 | ****** | 0.0083 | One way ANOVA followed by Tukey’s test |
| Fig.5H Vehicle (PDGF-BB) Vs Wort. (PDGF-BB) pFoxO3(Ser253)/ FoxO3 | ***** | 0.0137 | One way ANOVA followed by Tukey’s test |
| Fig.5H Vehicle (No PDGF-BB) Vs Vehicle (PDGF-BB) pFoxO3(Thr32)/ FoxO3 | ******* | 0.0004 | One way ANOVA followed by Tukey’s test |
| Fig.5H Vehicle (PDGF-BB) Vs Wort. (PDGF-BB) pFoxO3(Thr32)/ FoxO3 | ***** | 0.0109 | One way ANOVA followed by Tukey’s test |
| Fig.5I Vehicle (No PDGF-BB) Vs Vehicle (PDGF-BB) pAKT (Thr308)/AKT | ***** | 0.0214 | One way ANOVA followed by Tukey’s test |
| Fig.5I Vehicle (PDGF-BB) Vs Wort. (PDGF-BB) pAKT (Thr308)/AKT | ***** | 0.0218 | One way ANOVA followed by Tukey’s test |

**Fig. 6**

| **Groups compared** | **Summary** | **p value** | **Statistical test** |
| --- | --- | --- | --- |
| Fig.6A Vehicle (No TGFβ1) Vs Vehicle (TGFβ1) | ****** | 0.0035 | One way ANOVA followed by Tukey’s test |
| Fig.6A Vehicle (TGFβ1) Vs UCN-01 (TGFβ1) | ******* | <0.0001 | One way ANOVA followed by Tukey’s test |
| Fig.6C Vehicle (No TGFβ1) Vs Vehicle (TGFβ1) pFoxO3(Thr32)/ FoxO3 | ****** | 0.0033 | One way ANOVA followed by Tukey’s test |
| Fig.6C Vehicle (TGFβ1) Vs UCN-01 pFoxO3pFoxO3(Thr32)/ FoxO3 | ****** | 0.0038 | One way ANOVA followed by Tukey’s test |
| Fig.6C Vehicle (No TGFβ1) Vs Vehicle (TGFβ1) FoxO3/GAPDH | ***** | 0.0149 | One way ANOVA followed by Tukey’s test |
| Fig.6C Vehicle (TGFβ1) Vs UCN-01 FoxO3/GAPDH | ******* | 0.0004 | One way ANOVA followed by Tukey’s test |

**Fig. 7**

| **Groups compared** | **Summary** | **p value** | **Statistical test** |
| --- | --- | --- | --- |
| Fig.7A Scramble siRNA (5%FCS) Vs FoxO3 siRNA (5%FCS) | ****** | 0.027 | One way ANOVA followed by Tukey’s test |
| Fig.7B Scramble siRNA Vs Scramble siRNA- TGFβ1 COL1A1 | ****** | 0.027 | One way ANOVA followed by Tukey’s test |
| Fig.7B Scramble siRNA- TGFβ1 Vs Scramble siRNA- TGFβ1/UCN-01 COL1A1 | **§** | 0.0153 | One way ANOVA followed by Tukey’s test |
| Fig.7B Scramble siRNA- TGFβ1/UCN-01 Vs FoxO3 siRNA- TGFβ1/UCN-01 COL1A1 | **#** | 0.0315 | One way ANOVA followed by Tukey’s test |
| Fig.7B Scramble siRNA- TGFβ1 Vs Scramble siRNA- TGFβ1/UCN-01 COL3A1 | **§** | 0.0135 | One way ANOVA followed by Tukey’s test |

**Fig. 8**

| **Groups compared** | **Summary** | **p value** | **Statistical test** |
| --- | --- | --- | --- |
| Fig.8A Vehicle Vs UCN-01 (50nM) | ******* | <0.0001 | One way ANOVA followed by Tukey’s test |
| Fig.8A Vehicle Vs UCN-01 (100nM) | ******* | <0.0001 | One way ANOVA followed by Tukey’s test |
| Fig.8A Vehicle Vs UCN-01 (200nM) | ******* | <0.0001 | One way ANOVA followed by Tukey’s test |
| Fig.8A UCN-01 (50nM) Vs UCN-01 (100nM) | **§§§** | <0.0001 | One way ANOVA followed by Tukey’s test |
| Fig.8A UCN-01 (50nM) Vs UCN-01 (200nM) | **§§§** | <0.0001 | One way ANOVA followed by Tukey’s test |
| Fig.8A UCN-01 (100nM) Vs UCN-01 (200nM) | **##** | 0.0015 | One way ANOVA followed by Tukey’s test |
| Fig.8D Vehicle Bleo Vs Saline | ****** | 0.0038 | One way ANOVA followed by Tukey’s test |
| Fig.8D Vehicle Bleo Vs UCN-01 5mg/kg | ****** | 0.0048 | One way ANOVA followed by Tukey’s test |
| Fig.8D Vehicle Bleo Vs UCN-01 7.5mg/kg | ******* | <0.0001 | One way ANOVA followed by Tukey’s test |
| Fig.8E Vehicle Bleo Vs Saline | ******* | <0.0001 | One way ANOVA followed by Tukey’s test |
| Fig.8E Vehicle Bleo Vs UCN-01 5mg/kg | ***** | 0.0473 | One way ANOVA followed by Tukey’s test |
| Fig.8E Vehicle Bleo Vs UCN-01 7.5mg/kg | ****** | 0.0018 | One way ANOVA followed by Tukey’s test |
| Fig.8F Vehicle Bleo Vs Saline | ******* | <0.0001 | One way ANOVA followed by Tukey’s test |
| Fig.8F Vehicle Bleo Vs UCN-01 7.5mg/kg | ***** | 0.0171 | One way ANOVA followed by Tukey’s test |
| Fig.8H Vehicle Bleo Vs Saline | ******* | <0.0001 | One way ANOVA followed by Tukey’s test |
| Fig.8H Vehicle Bleo Vs UCN-01 5mg/kg | ***** | 0.0234 | One way ANOVA followed by Tukey’s test |
| Fig.8H Vehicle Bleo Vs UCN-01 7.5mg/kg | ******* | 0.0003 | One way ANOVA followed by Tukey’s test |

**Fig. 9**

| **Groups compared** | **Summary** | **p value** | **Statistical test** |
| --- | --- | --- | --- |
| Fig.9A WT Saline Vs WT Bleo/Vehicle | ******* | <0.0001 | One way ANOVA followed by Tukey’s test |
| Fig.9A WT Saline Vs Foxo3^-/-^ Bleo/Vehicle | ******* | <0.0001 | One way ANOVA followed by Tukey’s test |
| Fig.9A WT Saline Vs Foxo3^-/-^ Bleo/UCN-01 | ******* | <0.0001 | One way ANOVA followed by Tukey’s test |
| Fig.9A WT Bleo/Vehicle Vs Foxo3^-/-^ Bleo/Vehicle | **#** | 0.0191 | One way ANOVA followed by Tukey’s test |
| Fig.9A WT Bleo/UCN-01 Vs Foxo3^-/-^ Bleo/UCN-01 | **§§§** | <0.0001 | One way ANOVA followed by Tukey’s test |
| Fig.9B WT Saline Vs WT Bleo/Vehicle | ******* | <0.0001 | One way ANOVA followed by Tukey’s test |
| Fig.9B WT Saline Vs Foxo3^-/-^ Bleo/Vehicle | ******* | <0.0001 | One way ANOVA followed by Tukey’s test |
| Fig.9B WT Saline Vs WT Bleo/UCN-01 | ***** | 0.0177 | One way ANOVA followed by Tukey’s test |
| Fig.9B WT Saline Vs Foxo3^-/-^ Bleo/UCN-01 | ******* | <0.0001 | One way ANOVA followed by Tukey’s test |
| Fig.9C WT Saline Vs WT Bleo/Vehicle | ******* | <0.0001 | One way ANOVA followed by Tukey’s test |
| Fig.9C WT Saline Vs Foxo3^-/-^ Bleo/Vehicle | ******* | <0.0001 | One way ANOVA followed by Tukey’s test |
| Fig.9C WT Saline Vs Foxo3^-/-^ Bleo/UCN-01 | ****** | 0.0085 | One way ANOVA followed by Tukey’s test |
| Fig.9D WT Saline Vs WT Bleo/Vehicle | ******* | <0.0001 | One way ANOVA followed by Tukey’s test |
| Fig.9D WT Saline Vs Foxo3^-/-^ Bleo/Vehicle | ******* | <0.0001 | One way ANOVA followed by Tukey’s test |
| Fig.9D WT Saline Vs Foxo3^-/-^ Bleo/UCN-01 | ******* | 0.0004 | One way ANOVA followed by Tukey’s test |
| Fig. 9E EV (TGFβ1) Vs EV (TGFβ1)-UCN-01 50nM | ***** | 0.0151 | One way ANOVA followed by Tukey’s test |
| Fig. 9E EV (TGFβ1) Vs EV (TGFβ1)-UCN-01 100nM | ***** | 0.0100 | One way ANOVA followed by Tukey’s test |
| Fig.9F EV (TGFβ1) Vs EV (TGFβ1)-UCN-01 Col1A1 | ******* | <0.0001 | One way ANOVA followed by Tukey’s test |
| Fig.9F EV (TGFβ1)-UCN-01 Vs AKT mut (TGFβ1)-UCN-01 Col1A1 | **§** | 0.0134 | One way ANOVA followed by Tukey’s test |
| Fig.9F EV (TGFβ1) Vs EV (TGFβ1)-UCN-01 Col3A1 | ******* | 0.0004 | One way ANOVA followed by Tukey’s test |
| Fig.9F EV (TGFβ1)-UCN-01 Vs AKT mut (TGFβ1)-UCN-01 Col3A1 | **§** | 0.0130 | One way ANOVA followed by Tukey’s test |

**Fig. EV1**

| **Groups compared** | **Summary** | **p value** | **Statistical test** |
| --- | --- | --- | --- |
| Fig. EV1A No PDGF-BB (15min) Vs PDGF-BB(15min) 30ng/ml pFoxO3(Thr32)/FoxO3 | ****** | 0.0093 | One way ANOVA followed by Tukey’s test |
| Fig. EV1A No PDGF-BB (30min) Vs PDGF-BB(30min) 30ng/ml pFoxO3(Thr32)/FoxO3 | **§§§** | 0.0003 | One way ANOVA followed by Tukey’s test |
| Fig. EV1A No PDGF-BB (15min) Vs PDGF-BB(15min) 60ng/ml pFoxO3(Thr32)/FoxO3 | ****** | 0.0017 | One way ANOVA followed by Tukey’s test |
| Fig. EV1A No PDGF-BB (30min) Vs PDGF-BB(30min) 60ng/ml pFoxO3(Thr32)/FoxO3 | **§§** | 0.0030 | One way ANOVA followed by Tukey’s test |
| Fig. EV1A No PDGF-BB (15min) Vs PDGF-BB(15min) 30ng/ml pFoxO3(Ser253)/FoxO3 | ****** | 0.0066 | One way ANOVA followed by Tukey’s test |
| Fig. EV1A No PDGF-BB (30min) Vs PDGF-BB(30min) 30ng/ml pFoxO3(Ser253)/FoxO3 | **§** | 0.0173 | One way ANOVA followed by Tukey’s test |
| Fig. EV1A No PDGF-BB (15min) Vs PDGF-BB(15min) 60ng/ml pFoxO3(Ser253)/FoxO3 | ****** | 0.0036 | One way ANOVA followed by Tukey’s test |
| Fig. EV1A No PDGF-BB (30min) Vs PDGF-BB(30min) 60ng/ml pFoxO3(Ser253)/FoxO3 | **§§** | 0.0014 | One way ANOVA followed by Tukey’s test |

**Fig. EV3**

| **Groups compared** | **Summary** | **p value** | **Statistical test** |
| --- | --- | --- | --- |
| Fig. EV3D WT saline Vs WT Bleo | ******* | 0.0008 | One way ANOVA followed by Tukey’s test |
| Fig. EV3D WT saline Vs Foxo3^-/-^ Bleo | ******* | <0.0001 | One way ANOVA followed by Tukey’s test |
| Fig. EV3D WT saline Vs Foxo3_f.b_^-/-^ Bleo | ******* | <0.0001 | One way ANOVA followed by Tukey’s test |
| Fig. EV3D WT Bleo Vs Foxo3_f.b_^-/-^ Bleo | **§§** | 0.0151 | One way ANOVA followed by Tukey’s test |

**Fig. EV5**

| **Groups compared** | **Summary** | **p value** | **Statistical test** |
| --- | --- | --- | --- |
| Fig. EV5A EV (5%FCS) Vs EV (5%FCS)-UCN-01 50nM | ** | 0.0018 | One way ANOVA followed by Tukey’s test |
| Fig. EV5A (5%FCS) Vs EV (5%FCS)-UCN-01 100nM | ** | 0.0010 | One way ANOVA followed by Tukey’s test |
| Fig. EV5B AKTmut (FCS) Vs EV (UCN-01) | *** | <0.0001 | One way ANOVA followed by Tukey’s test |
| Fig. EV5B EV (UCN-01) Vs AKTmut  (UCN-01) | *** | <0.0001 | One way ANOVA followed by Tukey’s test |

**Fig. S2**

| **Groups compared** | **Summary** | **p value** | **Statistical test** |
| --- | --- | --- | --- |
| Fig. S2A No FCS (15min) Vs 5%FCS (15min) pFoxO3(Thr32)/FoxO3 | ****** | 0.0016 | One way ANOVA followed by Tukey’s test |
| Fig. S2A No FCS (30min) Vs 5%FCS (30min) pFoxO3(Thr32)/FoxO3 | **§§** | 0.0011 | One way ANOVA followed by Tukey’s test |
| Fig. S2A No FCS (15min) Vs 5%FCS (15min) pFoxO3(Ser253)/FoxO3 | ******* | <0.0001 | One way ANOVA followed by Tukey’s test |
| Fig. S2A No FCS (30min) Vs 5%FCS (30min) pFoxO3(Ser253)/FoxO3 | **§§§** | <0.0001 | One way ANOVA followed by Tukey’s test |

**Fig. S3**

| **Groups compared** | **Summary** | **p value** | **Statistical test** |
| --- | --- | --- | --- |
| Fig. S3A No IGF-1 (15min) Vs IGF-1 (15min) 100ng/ml pFoxO3(Thr32)/FoxO3 | ***** | 0.0160 | One way ANOVA followed by Tukey’s test |
| Fig. S3A No IGF-1 (30min) Vs IGF-1 (30min) 100ng/ml pFoxO3(Thr32)/FoxO3 | **§§§** | 0.0006 | One way ANOVA followed by Tukey’s test |
| Fig. S3A No IGF-1 (15min) Vs IGF-1 (15min) 200ng/ml pFoxO3(Thr32)/FoxO3 | ******* | 0.00045 | One way ANOVA followed by Tukey’s test |
| Fig. S3A No IGF-1 (30min) Vs IGF-1 (30min) 200ng/ml pFoxO3(Thr32)/FoxO3 | **§§§** | 0.0004 | One way ANOVA followed by Tukey’s test |
| Fig. S3A No IGF-1 (30min) Vs IGF-1 (30min) 100ng/ml pFoxO3(Ser253)/FoxO3 | **§** | 0.0139 | One way ANOVA followed by Tukey’s test |
| Fig. S3A No IGF-1 (15min) Vs IGF-1 (15min) 200ng/ml pFoxO3(Ser253)/FoxO3 | ***** | 0.0342 | One way ANOVA followed by Tukey’s test |
| Fig. S3A No IGF-1 (30min) Vs IGF-1 (30min) 200ng/ml pFoxO3(Ser253)/FoxO3 | **§** | 0.0126 | One way ANOVA followed by Tukey’s test |

**Fig. S4**

| **Groups compared** | **Summary** | **p value** | **Statistical test** |
| --- | --- | --- | --- |
| Fig. S4 Vehicle Vs 5%FCS | ***** | 0.0421 | Student’s t test |
| Fig. S4 Vehicle Vs PDGF-BB | ***** | 0.0318 | Student’s t test |

**Fig. S5**

| **Groups compared** | **Summary** | **p value** | **Statistical test** |
| --- | --- | --- | --- |
| Fig. S5 Vehicle Vs 5%FCS | ******* | 0.0002 | Student’s t test |
| Fig. S5 Vehicle Vs PDGF-BB | ***** | 0.0260 | Student’s t test |

**Fig. S7**

| **Groups compared** | **Summary** | **p value** | **Statistical test** |
| --- | --- | --- | --- |
| Fig. S7 No TGFβ1 (15min) Vs TGFβ1 (15min) 10ng/ml pFoxO3(Thr32)/FoxO3 | ****** | 0.0019 | One way ANOVA followed by Tukey’s test |
| Fig. S7 No TGFβ1 (30min) Vs TGFβ1 (30min) 10ng/ml pFoxO3(Thr32)/FoxO3 | **§** | 0.0110 | One way ANOVA followed by Tukey’s test |

**Fig. S10**

| **Groups compared** | **Summary** | **p value** | **Statistical test** |
| --- | --- | --- | --- |
| Fig. S10 Scramble siRNA Vs FoxO3 siRNA | ***** | 0.0254 | Student’s t Test |

**Fig. S11**

| **Groups compared** | **Summary** | **p value** | **Statistical test** |
| --- | --- | --- | --- |
| Fig. S11 WT littermates Vs Foxo3_f.b_ ^-/-^ | ***** | 0.0141 | Student’s t Test |

**Fig. S13**

| **Groups compared** | **Summary** | **p value** | **Statistical test** |
| --- | --- | --- | --- |
| Fig. S13 5% FCS cyto Vs UCN-01 nuclear | ***** | 0.0260 | Student’s t Test |
| Fig. S13 UCN-01 cyto Vs UCN-01 nuclear | ***** | 0.0151 | Student’s t Test |

**Fig. S14**

| **Groups compared** | **Summary** | **p value** | **Statistical test** |
| --- | --- | --- | --- |
| Fig. S14 Vehicle Vs TGFβ1 | ***** | 0.0252 | One way ANOVA followed by Tukey’s test |
| Fig. S14 TGFβ1 Vs Wort. (250nM) | ****** | 0.0011 | One way ANOVA followed by Tukey’s test |
| Fig. S14 TGFβ1 Vs Wort. (500nM) | ******* | 0.0007 | One way ANOVA followed by Tukey’s test |
| Fig. S14 TGFβ1 Vs UCN-01 (50nM) | ******* | 0.0005 | One way ANOVA followed by Tukey’s test |
