# Supplementary material for: FoxO3 an important player in fibrogenesis and therapeutic target for idiopathic pulmonary fibrosis
Source: EMBO Mol Med. 2017 Dec 7;10(2):276–93. doi: 10.15252/emmm.201606261 (PMC5801513; doi:10.15252/emmm.201606261)
Supplement: Supplementary file 2 — Expanded View Figures PDF [file EMMM-10-276-s002.pdf]

Expanded View Figures

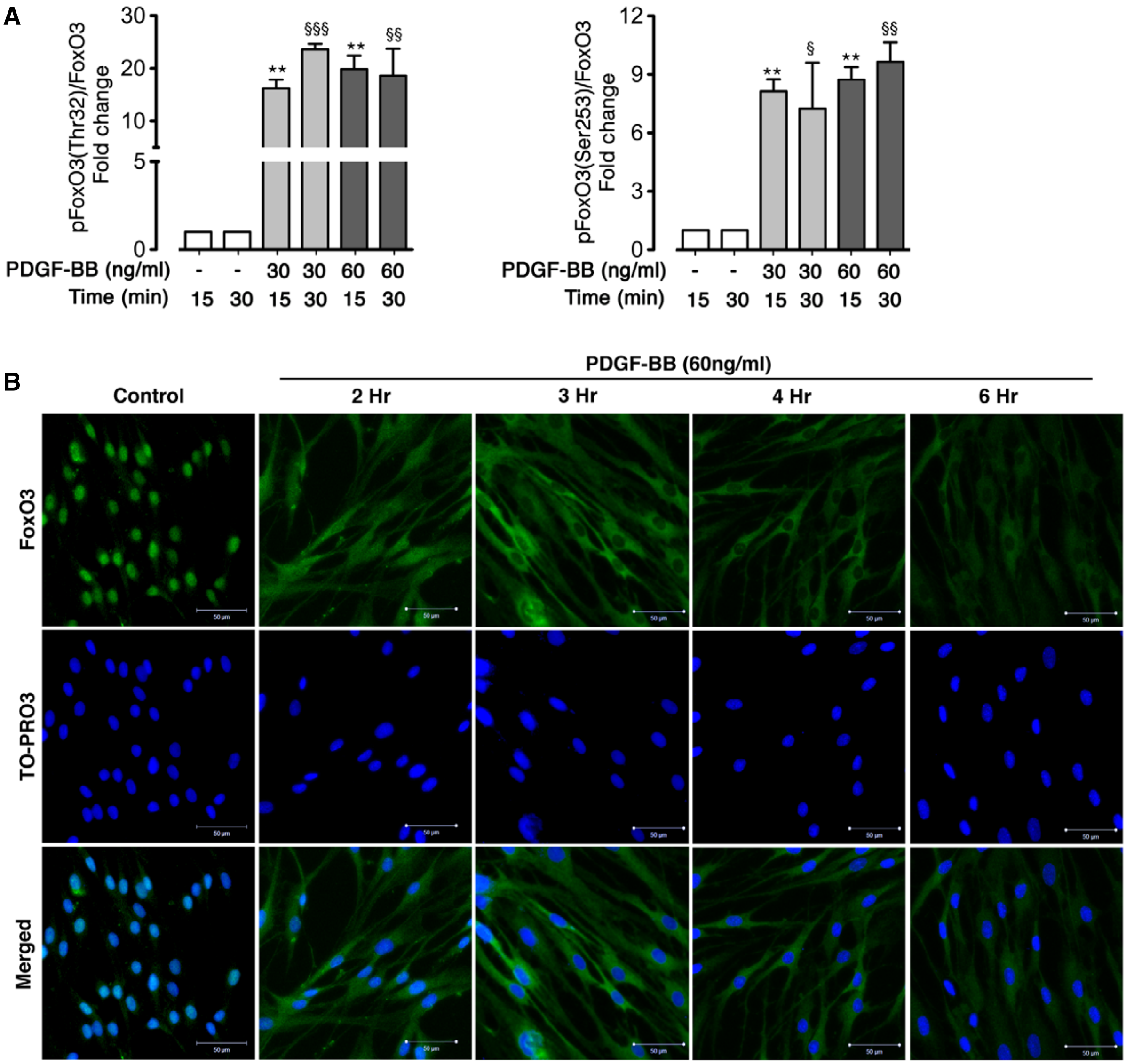

Figure EV1. PDGF induces FoxO3 phosphorylation and nuclear exclusion of FoxO3.

A Densitometry quantitation ratio of p-FoxO3 (Thr32) (left panel) and p-FoxO3 (Ser253) (right panel) in serum-starved (48 h) N-HLF (n = 3) that were stimulated without/with PDGF-BB as indicated. Quantification is represented as a fold change to control (time corresponded non-stimulated cells). Data were analyzed using repeated-measures ANOVA, $^{**}P < 0.01$ versus control 15 min, $^{\S}P < 0.05$, $^{\S\S}P < 0.01$, $^{\S\S\S}P < 0.001$ versus control 30 min.

B ICC of FoxO3 in N-HLF that was serum-starved for 48 h, and stimulated with PDGF-BB (60 ng/ml) as indicated. Control image panel represents cells that were left non-stimulated for 6 h. TO-PRO3 (blue) was used to label nuclei. FoxO3 and TO-PRO3 images were overlaid to visualize nuclear and cytoplasmic localization of FoxO3. Images are representative of n = 3. Scale bar = 50 µm.

© 2017 The Authors

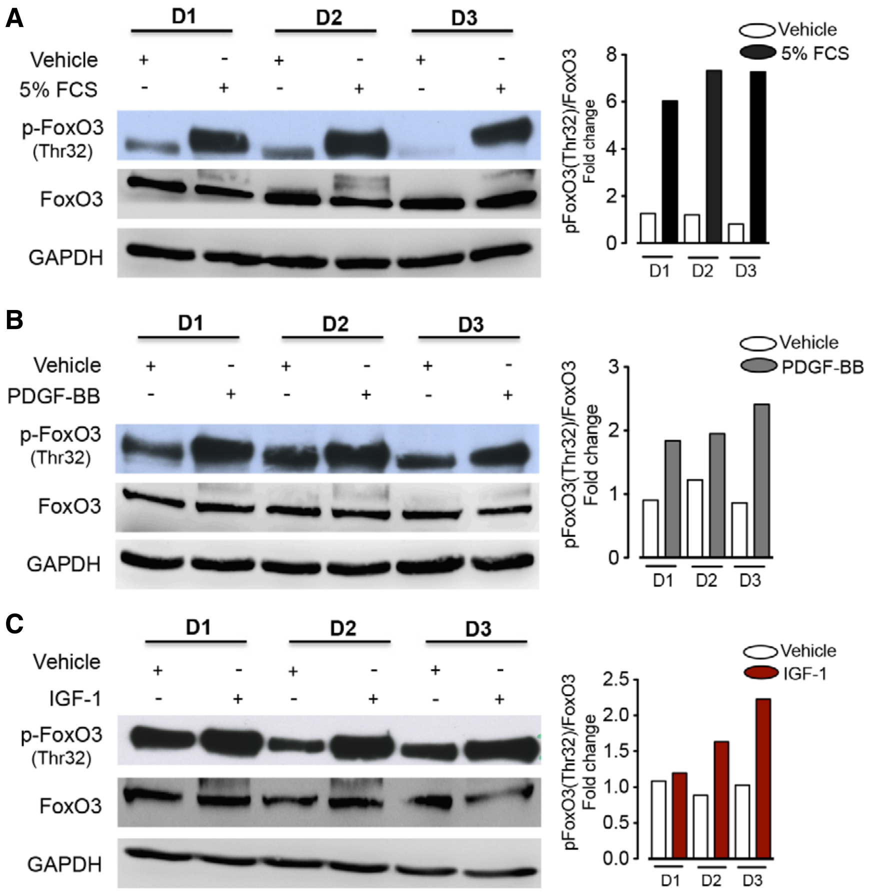

Figure EV2. Different N-HLFs respond in similar manner to various growth factors.

A–C Western blots of p-FoxO3 (Thr32), FoxO3, and GAPDH in serum-starved (48 h) N-HLF (n = 3) that was stimulated with 5% FCS (A), PDGF-BB (B), or IGF-1 (C) as indicated. Densitometry quantified data of p-FoxO3 (Thr32) to FoxO3 expression ratios, represented as a fold change to non-stimulated cells.

Figure EV3. Foxo3 knockout (global- and fibroblast-specific) influences immune cell composition in bleomycin-instilled mice lungs.

A–D Immunofluorescence staining was performed on WT, *Foxo3*$^{-/-}$ and *Foxo3*$_{f.b}^{-/-}$ mice lung sections (saline- and bleomycin-instilled) using CD68, CD45, and CD3 antibodies. Representative pictographs depicting CD68 (A), CD45 (B), and CD3 (C) staining in green from mice (n = 3) in each group. DAPI was used as a nuclear stain. Scale bar = 50 μm. Fluorescence intensities of CD3-stained sections (n = 5/6 per group) were quantified using ImageJ software and normalized to DAPI intensity. Data are expressed as mean ± SEM and were analyzed using repeated-measures one-way ANOVA, ***P < 0.001 versus WT saline group and $^{\S\S}P$ < 0.01 versus WT bleomycin group. m1, m2, and m3 represent three different mice evaluated in each group.

 © 2017 The Authors

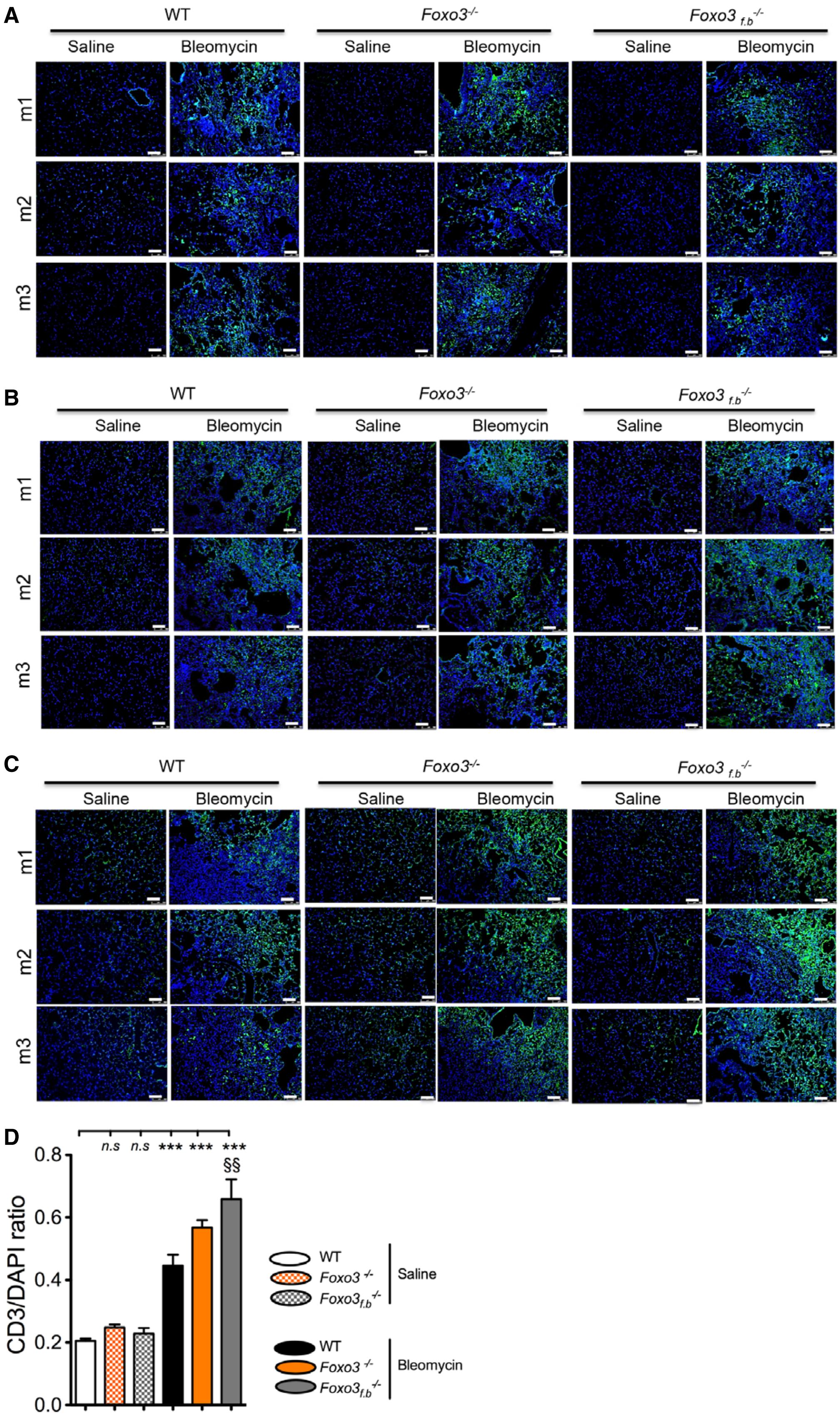
A
WT
Foxo3-/-
Foxo3 f.b-/-
Saline
Bleomycin
m1
m2
m3
B
C
D
n.s
n.s
***
***
***
§§
CD3/DAPI ratio
0.0
0.2
0.4
0.6
0.8
WT
Foxo3 -/-
Foxo3f.b-/-
Saline
Bleomycin

Figure EV3.

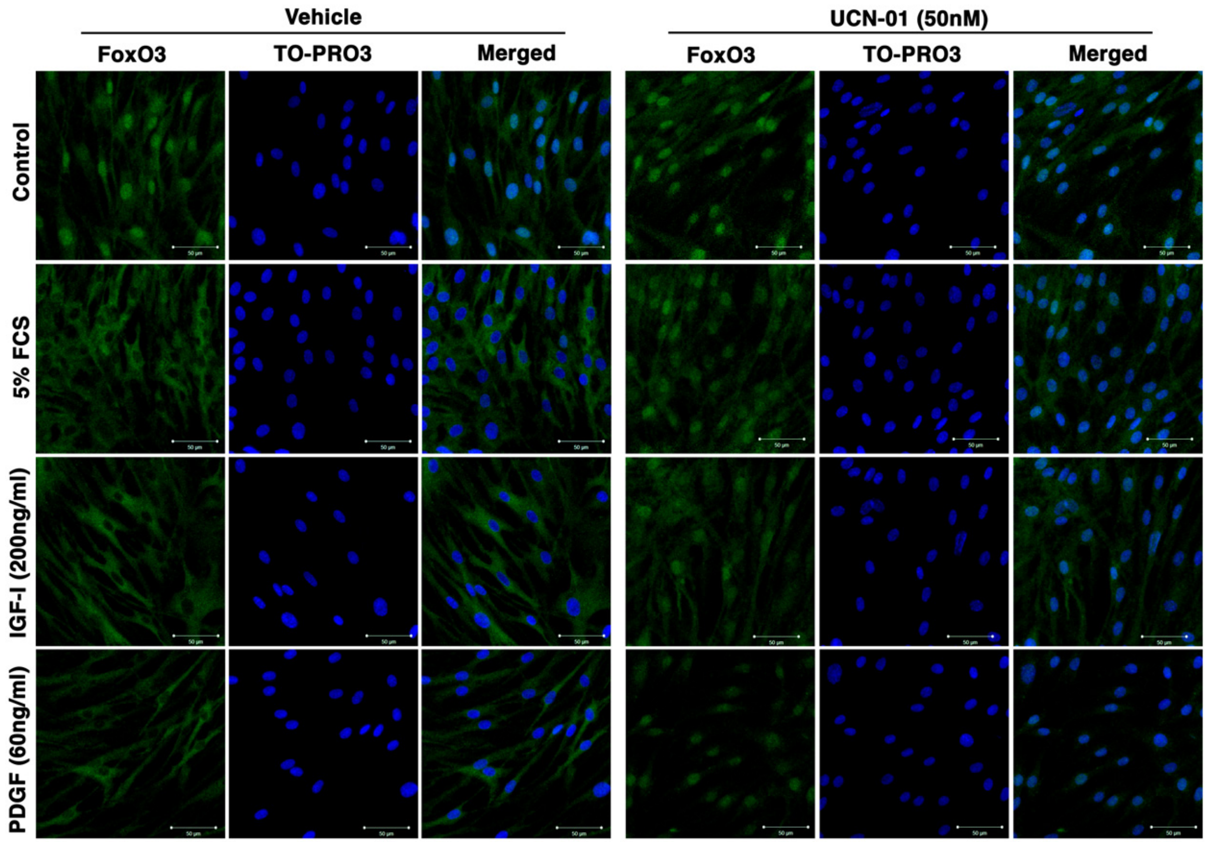

Figure EV4. UCN-01 inhibits FoxO3 nuclear exclusion of FCS-, IGF-1- or PDGF-BB- stimulated IPF-HLF.

Serum-starved (48 h) IPF-HLF were stimulated with 5% FCS or PDGF-BB (60 ng/ml) or IGF-1 (200 ng/ml) and treated with 50 nM UCN-01 or vehicle control (DMSO). After 6 h of treatment, ICC assessed FoxO3 cellular localization. Control panel represents cells that were treated for 6 h with 50 nM UCN-01 or vehicle control (DMSO). TO-PRO3 (blue) was used to label nuclei. FoxO3 and TO-PRO3 images were overlaid to visualize nuclear and cytoplasmic localization of FoxO3. Scale bar = 50 μm. Images are representative of $n = 3$.

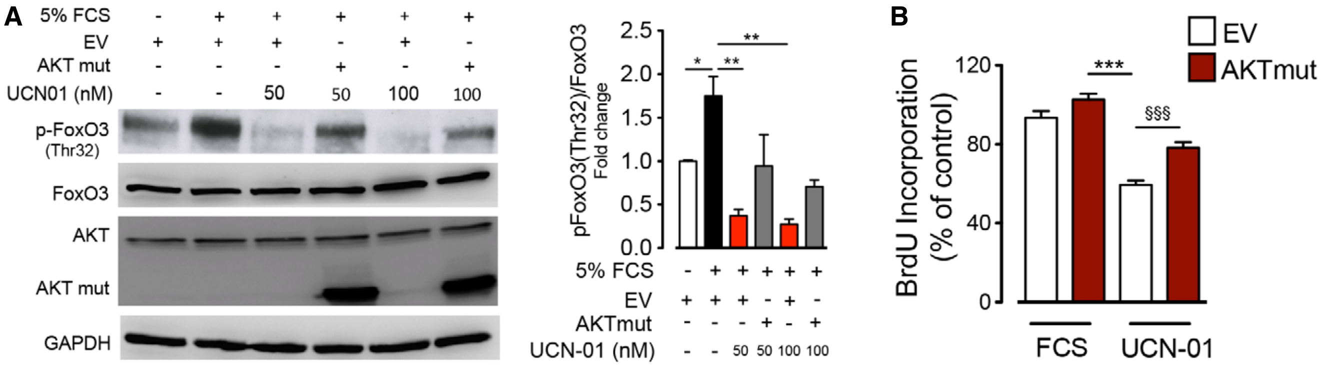

Figure EV5. Anti-proliferative effects of UCN-01 are mediated via FoxO3.

A, B N-HLF was transfected with empty vector (EV) or AKT mutant (AKT mut) plasmid. 6 h after transfection, cells were serum-starved for 36 h and then stimulated with 5% FCS in the presence or absence of UCN-01. From the above-treated samples, after 24 h, Western blots [p-FoxO3 (Thr32), FoxO3, AKT, AKT mut, GAPDH] and cell proliferation measurements (BrdU incorporation) were performed. Data represent percentage of control, EV non-stimulated cells ($n = 3$). Data are expressed as mean ± SEM and were analyzed using one-way ANOVA, $*P < 0.05$, $**P < 0.01$, and $***P < 0.001$ versus 5% FCS-EV, and $^{§§§}P < 0.001$ versus UCN-01-EV.

 © 2017 The Authors
