# Supplementary figures and images for: FoxO3 an important player in fibrogenesis and therapeutic target for idiopathic pulmonary fibrosis
Source: EMBO Mol Med. 2017 Dec 7;10(2):276–93. doi: 10.15252/emmm.201606261 (PMC5801513; doi:10.15252/emmm.201606261)

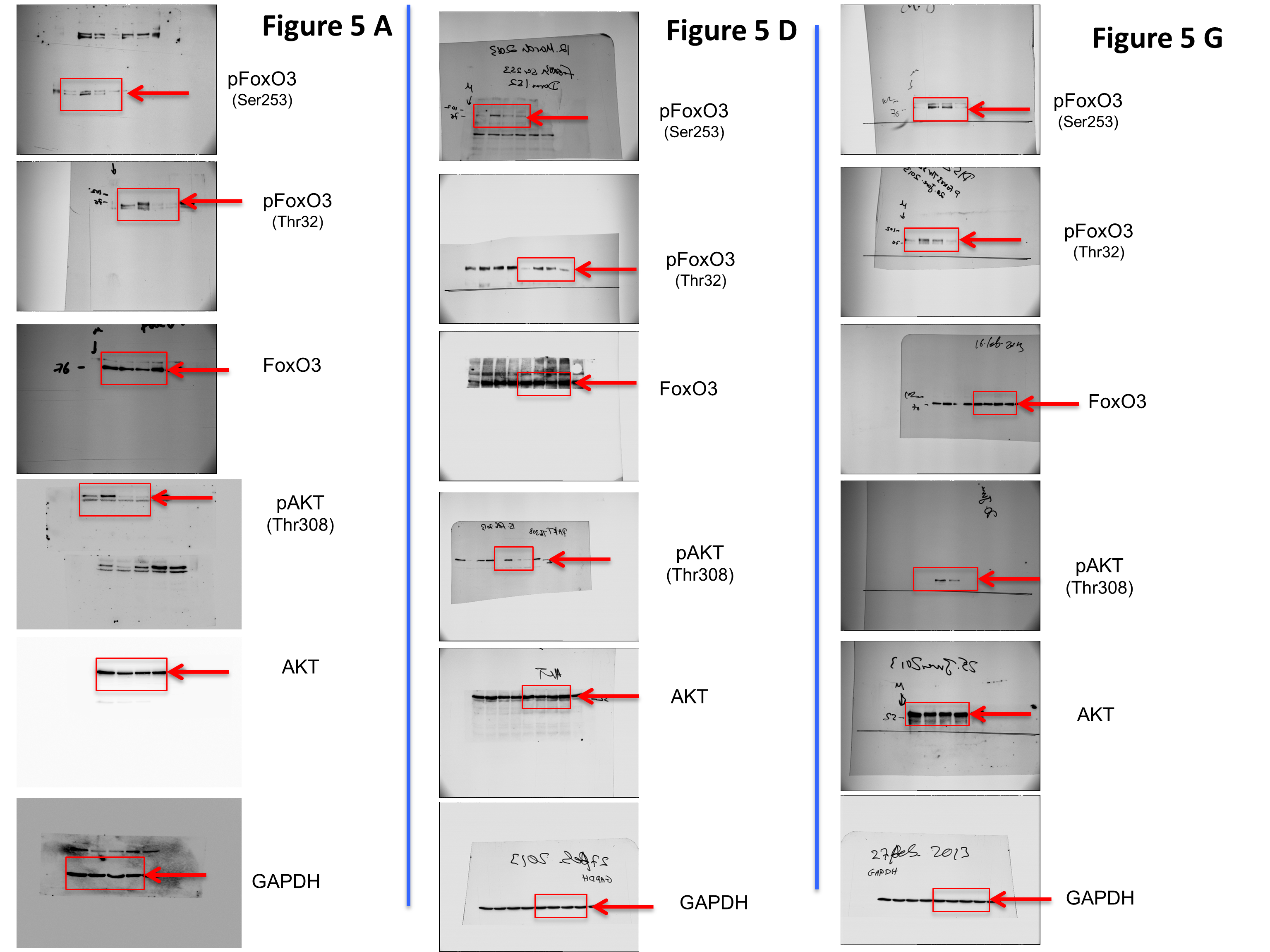

Supplement: Supplementary file 4 — Source Data for Figure 5 [file EMMM-10-276-s003.TIF]

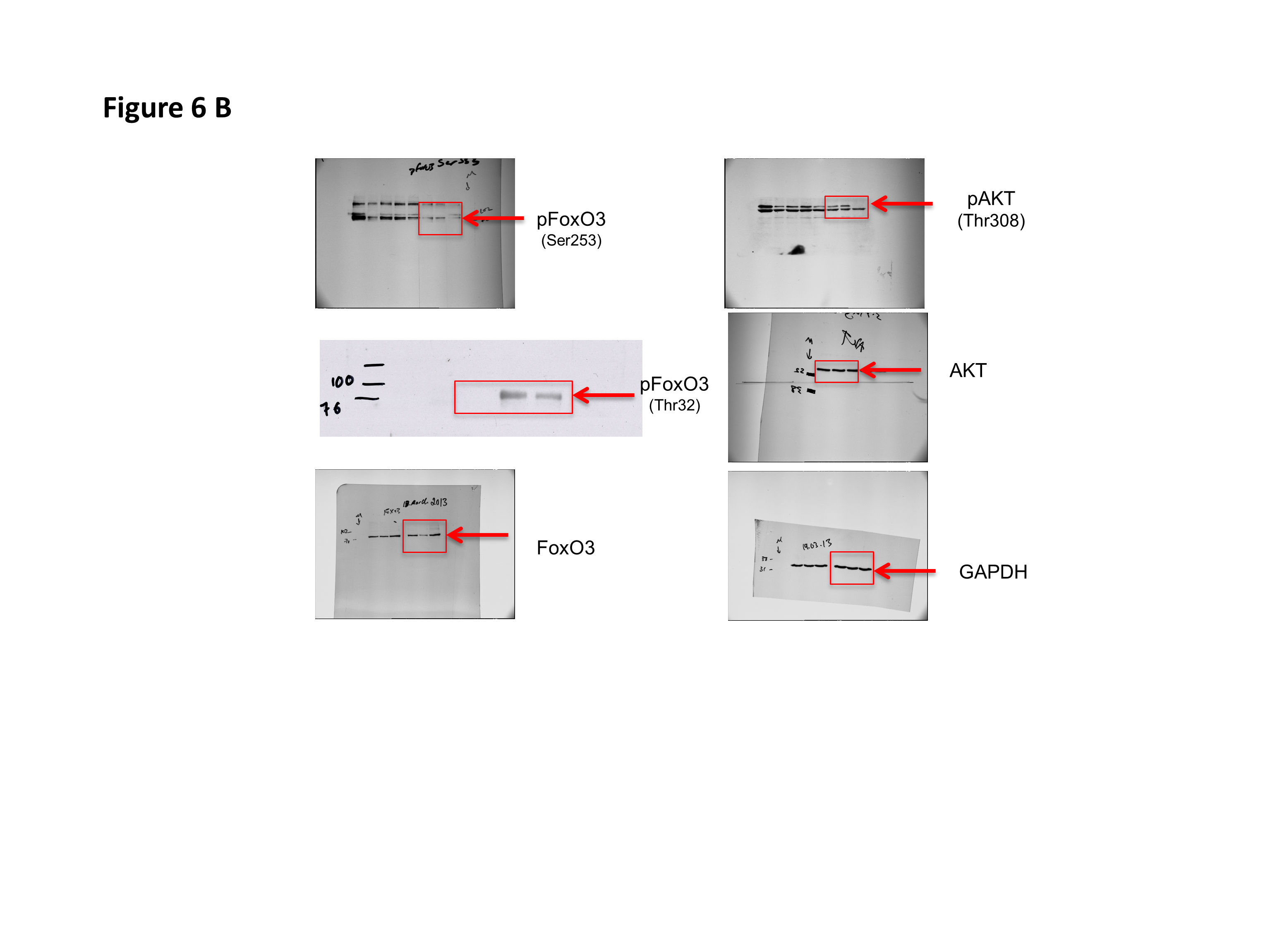

Supplement: Supplementary file 5 — Source Data for Figure 6 [file EMMM-10-276-s004.TIF]

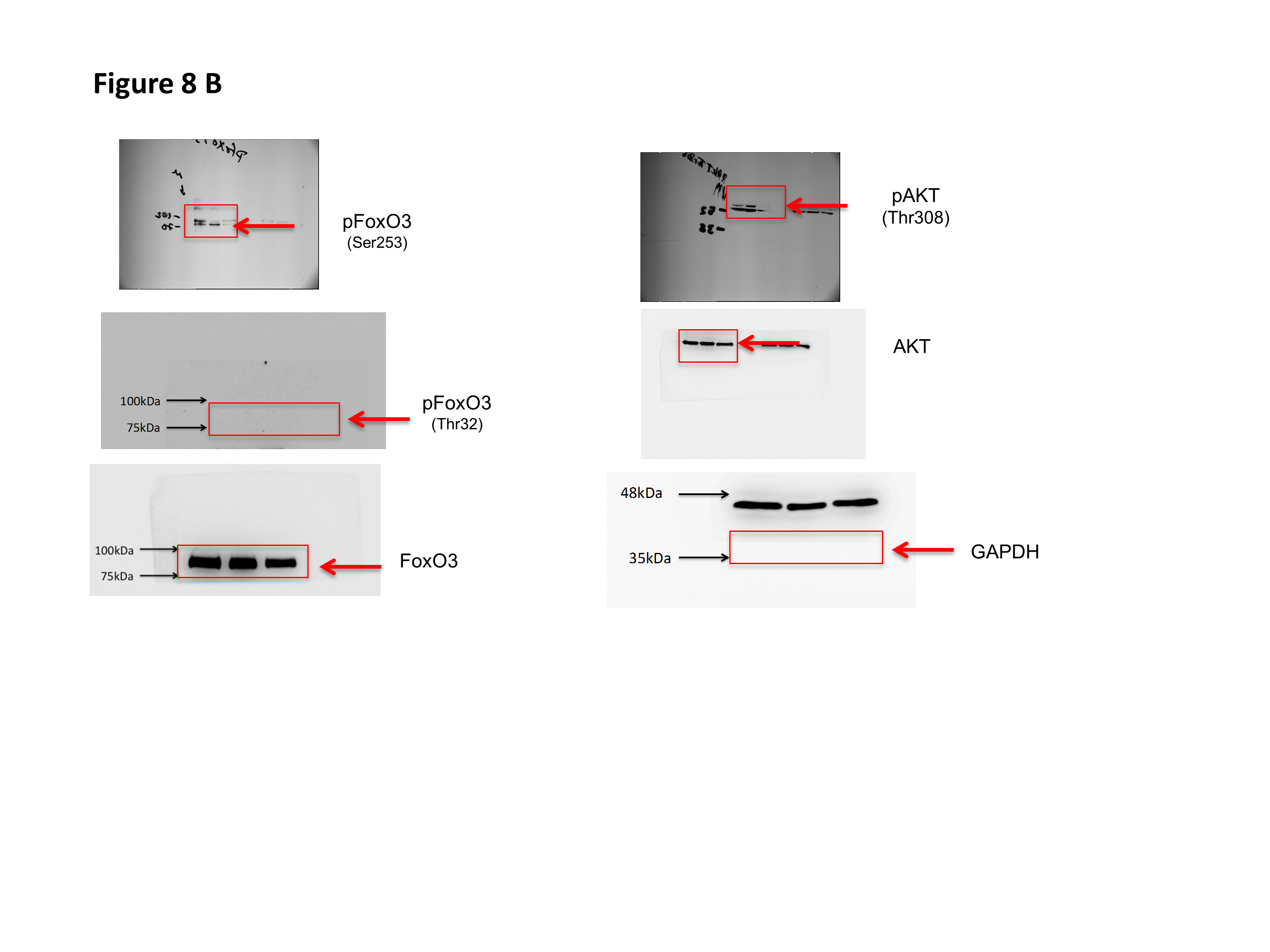

Supplement: Supplementary file 6 — Source Data for Figure 8 [file EMMM-10-276-s005.TIF]
